# Stem cells in articular cartilage regeneration

**DOI:** 10.1186/s13018-016-0378-x

**Published:** 2016-04-12

**Authors:** Giuseppe Filardo, Francesco Perdisa, Alice Roffi, Maurilio Marcacci, Elizaveta Kon

**Affiliations:** II Orthopaedic and Traumatologic Clinic, Rizzoli Orthopaedic Institute, Bologna, Italy; Nanobiotechnology Laboratory, Rizzoli Orthopaedic Institute, Via di Barbiano 1/10, 40136 Bologna, Italy

## Abstract

Mesenchymal stem cells (MSCs) have emerged as a promising option to treat articular defects and early osteoarthritis (OA) stages. However, both their potential and limitations for a clinical use remain controversial. Thus, the aim of this systematic review was to examine MSCs treatment strategies in clinical settings, in order to summarize the current evidence of their efficacy for the treatment of cartilage lesions and OA.

Among the 60 selected studies, 7 were randomized, 13 comparative, 31 case series, and 9 case reports; 26 studies reported the results after injective administration, whereas 33 used surgical implantation. One study compared the two different modalities. With regard to the cell source, 20 studies concerned BMSCs, 17 ADSCs, 16 BMC, 5 PBSCs, 1 SDSCs, and 1 compared BMC versus PBSCs. Overall, despite the increasing literature on this topic, the evidence is still limited, in particular for high-level studies. On the other hand, the available studies allow to draw some indications. First, no major adverse events related to the treatment or to the cell harvest have been reported. Second, a clinical benefit of using MSCs therapies has been reported in most of the studies, regardless of cell source, indication, or administration method. This effectiveness has been reflected by clinical improvements and also positive MRI and macroscopic findings, whereas histologic features gave more controversial results among different studies. Third, young age, lower BMI, smaller lesion size for focal lesions, and earlier stages of OA joints have been shown to correlate with better outcomes, even though the available data strength does not allow to define clear cutoff values. Finally, definite trends can be observed with regard to the delivery method: currently cultured cells are mostly being administered by i.a. injection, while one-step surgical implantation is preferred for cell concentrates. In conclusion, while promising results have been shown, the potential of these treatments should be confirmed by reliable clinical data through double-blind, controlled, prospective and multicenter studies with longer follow-up, and specific studies should be designed to identify the best cell sources, manipulation, and delivery techniques, as well as pathology and disease phase indications.

## Background

Articular cartilage lesions are a debilitating disease, often resulting in fibrillation and subsequent degradation of the surrounding articular surface, possibly involving the subchondral bone as well, thus favoring the development of osteoarthritis (OA). OA affects up to 15 % of the adult population and represents the second greatest cause of disability worldwide [1], with a massive impact on society both in terms of quality of life for the individuals and high costs for the healthcare system [2]. Several approaches have been proposed for the management of cartilage degeneration, ranging from pharmacological to surgical options, aimed at reducing symptoms and restoring a satisfactory knee function [3, 4]. However, none of them has clearly shown the potential of restoring chondral surface and physiological joint homeostasis in order to prevent OA, which in the final stage often requires prosthetic replacement.

Among the solutions proposed to delay the need for metal resurfacing of the damaged articular surface, mesenchymal stem cells (MSCs) have recently emerged as a promising option to treat articular defects and early OA stages [5]. MSCs are multipotent progenitor cells that can differentiate into selected lineages including chondrocytes, with capability of self-renewal, high plasticity, and immunosuppressive and anti-inflammatory action [6, 7]. Moreover, Caplan and colleagues [8] recently underlined that these cells, derived from perivascular cells called “pericytes”, have a key role in the response to tissue injuries not just by differentiating themselves, but also by inducing repair/regeneration processes at the injury site through the secretion of several bioactive molecules [9]. In light of these properties, MSCs represent an excellent candidate for cell therapies and their healing potential has been explored also in terms of cartilage tissue regeneration and OA processes modulation [6]. The first investigations involved MSCs derived from bone marrow, which have been applied either as a cell suspension after being expanded by culture (BMSCs), or used as a simple bone marrow concentrate (BMC), thanks to their relative abundance [6]. Despite an extensive preclinical research and promising clinical results, some drawbacks related to the cell harvest and culture led to the development of different alternative options, with stem cells derived from adipose tissue (ADSCs), synovial tissue (SDSCs), and peripheral blood (PBSCs) [10, 11]. Besides these sources already explored and reported in the clinical use, cells derived from fetal tissues are being currently investigated at preclinical level [12]. Although numerous advancements have been made, the understanding of MSCs mechanism of action as well as their potential and limitations for the clinical use remain controversial. Many questions are still open on the identification of patients who might benefit more from this kind of treatment, as well as the most suitable protocol of administration (no. of cells, concentrated or culture-expanded, best harvest source, etc.).

Based on these premises, the aim of this systematic review was to examine the literature on MSCs treatment strategies in the clinical setting, in order to summarize the current evidence on their potential for the treatment of cartilage lesions and OA.

## Materials and methods

A systematic review of the literature was performed on the PubMed database by two independent reviewers using the following string: “cartilage treatment” AND “mesenchymal stem cells”. The filters included publications on the use of MSCs for cartilage defects and OA in the clinical field and in English language, published from 2000 to the end of 2015. Articles were first screened by title and abstract. Subsequently, the full texts of the resulting articles were screened and those not reporting clinical results of MSCs for cartilage and OA treatment were excluded. The reference lists of the selected articles were also screened to obtain further studies for this review.

## Results

**Fig. 1:**
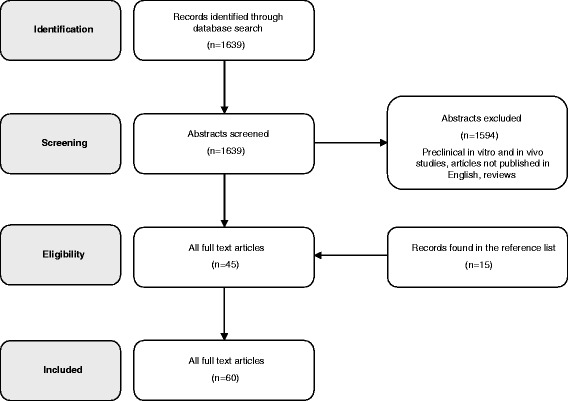
Scheme of research methodology

**Fig. 2:**
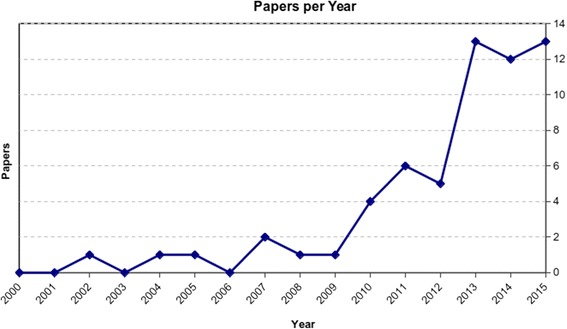
The systematic research showed an increasing number of clinical studies published over time

Our search identified 1639 papers after the screening process, 60 were included in the analysis (Fig. 1), which showed an increasing number of studies published on this topic over time (Fig. 2). Among the 60 selected studies, 7 were randomized, 13 comparative, 31 case series, and 9 case reports; 26 studies reported the results after injective administration, whereas 33 used surgical implantation. One study compared the two different modalities. With regard to the cell source, 20 studies concerned BMSCs, 17 ADSCs, 16 BMC, 5 PBSCs, 1 SDSCs, and 1 compared BMC versus PBSCs. While all the included studies are summarized in detail in Table 1 according to cell source and treatment strategy, the most relevant findings will be discussed in the following paragraphs.

**Table 1:** Details of the 60 clinical trials identified by the systematic review focusing on MSCs use for the treatment of cartilage pathology

MSCs	Publication	Study type	Treatment	Additional information	Pathology	*N* patients	Follow-up	Results
Cultured BMSCs	Davatchi [22] 2015 Int Journal of Rheum Disease	Case series	IA injection	Previous study update	Knee OA	3	60 months	Still significant improvement at 5 years, but gradual worsening after 6-month follow-up
	Vega [26] 2015 Transplantation	RCT	IA injection	Allogeneic BMSCs	Knee OA	15 BMSCs 15 HA	12 months	Significant better functional and cartilage quality improvements in MSCs group vs. control
	Sol Rich [18] 2015 J Stem Cell Res Ther	Case series	IA injection		Knee OA	12	24 months	Excellent clinical and quantitative MRI outcome measures at 2 years
	Vangsness [25] 2014 JBJS Am	RCT	IA injection	Allogeneic BMSCs After medial meniscectomy	Knee OA	18 low-dose MSCs + HA 18 high-dose MSCs + HA 19 HA	24 months	Knee pain improvement and evidence of meniscus regeneration at MRI for both doses vs. control
	Orozco [21] 2014 Transplantation	Case series	IA injection	Previous study update	Knee OA	12	24 months	Pain improvement at 12 months maintained at 24 months. The quality of cartilage further improved at MRI at 24 months
	Wong [24] 2013 Arthroscopy	RCT	IA injection	Comb HTO + MFX and post-op injection	Knee OA	28 BMSCs + HA 28 HA	24 months	BMSCs i.a. injection produced superior clinical and MRI outcomes at 24 months
	Ricther [35] 2013 Foot & Ankle	Case series	Surgical delivery	MAST Collagen membrane	Ankle chondral defects	25	24 months	No adverse events. Clinical scores improvement Positive findings at histology
	Orozco [22] 2013 Transplantation	Case series	IA injection		Knee OA	12	12 months	No safety issues. Rapid and progressive clinical improvement at 12 months 11/12 patients increased cartilage quality at MRI
	Lee [23] 2012 Ann Accad Med Singapore	Comparative	IA injection		Knee cartilage defects	35 MFX + BMSCs + HA 35 BMSCs + periosteal patch	24 months	MFX + BMSCs + HA had comparable results vs. BMSCs + periosteal patch, but lower invasivity
	Emadedin [19] 2012 Arch Iran Med	Case series	IA injection		Knee OA	6	12 months	No local or systemic adverse events. Decreased pain, improved function and walking distance 3/6 increased cartilage thickness at MRI
	Kasemkijwattana [29] 2011 J Med Assoc Thai	Case report	Surgical delivery	MAST Collagen membrane	Knee cartilage defects	2	31 months	Significant clinical improvement Good filling, tissue stiffness, and integration at 2nd look
	Davatchi [17] 2011 Int J Rheum Dis	Case series	IA injection		Knee OA	4	12 months	Encouraging clinical results no X-Rays improvement
	Haleem [28] 2010 Cartilage	Case series	Surgical delivery	MAST PRF as scaffold	Knee cartilage defects	5	12 months	5/5 symptoms improvement Complete defect filling and surface congruity with native cartilage in 3/5 at MRI
	Nejadnik [34] 2010 AJSM	Comparative	Surgical delivery	BMSCs + periosteal flap	Knee cartilage defects	36 ACI 36 BMSCs + periosteal flap	24 months	Comparable improvement in quality of life, health, and sport activity. M better than F, older than 45 years lower improvement only in ACI group.
	Centeno [16] 2008 Pain Physician	Case report	IA injection		Knee cartilage defects	1 IA BMSCs + 2 weekly platelet lysate IA injections	24 months	Improvement of range of motion and pain scores. Significant cartilage and meniscus growth at MRI
	Kuroda [30] 2007 Osteoarthritis & Cartilage	Case report	Surgical delivery	BMSCs + collagen gel + periosteum	Knee cartilage defects	1	12 months	Hyaline-like tissue regeneration, improvement in clinical symptoms and return to previous activity level
	Wakitani [31] 2007 J Tissue Eng Regen Med	Case report	Surgical delivery	BMSCs + collagen gel + periosteum or synovium	Knee cartilage defect patella	3	17–27 months	Improvement in clinical symptoms maintained over time. Fibrocartilaginous tissue at histology
	Adachi [27] 2005 J Rheumatol	Case report	Surgical delivery	MAST Hydroxyapatite ceramic	Knee osteochondral defect	1		Cartilage-like and bone tissue regeneration at 2nd look arthroscopy
	Wakitani [32] 2004 Cell Transplant	Case report	Surgical delivery	BMSCs + collagen gel + periosteum	Knee cartilage defect Patella	2	5 years	Short-term clinical improvement, then stable at 24 months fibrocartilage defect filling
	Wakitani [33] 2002 Osteoarthritis & Cartilage	Comparative	Surgical delivery	Collagen gel sheet + periosteum	Knee OA	12 BMSCs + HTO 12 cell-free control + HTO	16 months	Comparable clinical outcomes, but better arthroscopic and histological score in cell-transplanted group
BM Concentrate	Gobbi [47] 2015 Cartilage	Comparative	Surgical delivery	MAST HA matrix	Knee cartilage defects patellofemoral	19 MACT 18 BMC	3 years	Significant scores improvement in both groups. Better IKDC subj for BMC. MACI: trochlea better than patella; BMC: site n.s. Better filling at MRI for BMC
	Buda [40] 2015 Arch Orthop Trauma Surg	Case series	Surgical delivery	MAST HA matrix	OLTs and ankle OA	56	36 months	Clinical outcome improvement at 12 months, further increase at 24 months and lowering trend at 36 months Higher BMI and OA degree had worse results
	Buda [39] 2015 Cartilage	Case series	Surgical delivery	MAST HA matrix	Ankle osteochondral lesions (hemophilic patients)	5	24 months	Clinical improvement at 2 years. 3 patients back to sports. Signs of cartilage and bone tissue regeneration at MRI. No radiographic joint degeneration progression
	Buda R [43] 2015 Int Orthop	Comparative	Surgical delivery	MAST HA matrix + PRF	OLTs	40 ACI 40 BMC	48 months	ACI and MAST was equally effective for the treatment of OLT. MAST preferred for the 1 step procedure, and lower costs
	Gobbi [50] 2014 AJSM	Case series	Surgical delivery	MAST Collagen membrane	Knee chondral defects	25	3 years	Significant scores improvement Older than 45 and smaller or single lesions showed better outcomes. Good implant stability and complete filling at MRI.
	Cadossi [44] 2014 Foot Ankle Int	RCT	Surgical delivery	MAST HA matrix	OLTs	15 BMDCs + HA + PEMF 15 BMDCs + HA	12 months	Biophysical stimulation started soon after surgery aided patient recovery leading to pain control and a better clinical outcome with these improvements lasting more than 1 year after surgery
	Buda [41] 2014 Joints	Case series	Surgical delivery	MAST HA/collagen powder matrix + PRF	OLTs	41 BMAC + HA + PRF 23 BMAC + collagen powder + PRF	53 months	Significant clinical improvement, gradual decrease after 24+ months
	Skowronski [51] 2013 Orthop Traumatol Rehabil	Case series	Surgical delivery	MAST Collagen membrane	Knee chondral defects	54	5 years	Improvement in clinical scores in 52/54 patients without complications After 5 years n.s. deterioration in 3 patients
	Giannini [38] 2013 AJSM	Case series	Surgical delivery	MAST HA membrane or collagen powder + PRF	OLTs	49	24–48 months	Good clinical results at 24 months, then significant decrease at 36 and 48 months. T2 mapping similar to native hyaline cartilage and correlate with the clinical results
	Buda [46] 2013 Muskuloskeletal Surg	Case series	Surgical delivery	MAST HA matrix	OLKs	30	29 months	Good clinical outcome osteochondral regeneration at control MRI and biopsies
	Gigante [48] 2012 Arhtroscopy Technique	Case report	Surgical delivery	MAST Collagen membrane + MFX	Knee chondral defects	1	24 months	Pain free at 6 months, still asymptomatic at 24 months Positive MRI tissue appearance at 12 months
	Gigante [49] 2011 Int J Immunopathol Pharmacol	Case series	Surgical delivery	MAST collagen membrane	Knee chondral defects	5	12 months	Patients asymptomatic Nearly normal arthroscopic appearance and satisfactory repair tissue at 12 months
	Giannini [42] 2010 Injury	Comparative	Surgical delivery	MAST HA matrix + PRF	OLTs	10 ACI open 46 arthroscopic MACT 25 arthroscopic MAST	36 months	Similar clinical improvement among groups. Good restoration of the cartilaginous layer with hyaline-like characteristics at MRI and histology
	Varma [36] 2010 J Indian Med Assoc	Comparative	IA injection	Augmentation to debridement	Knee OA	25 Debridement + BMC 25 Debridement alone	6 months	BMC: higher improvement in symptoms, function, and quality of life
	Buda [45] 2010 JBJS Am	Case series	Surgical delivery	MAST HA matrix + PRF	OLKs	20	24 months	Significant clinical improvement at 12 and 24 months. Associated procedures delayed recovery. Satisfactory MRI findings in 80 % of patients
	Giannini [37] 2009 Clin Orthop Rel Res	Case series	Surgical delivery	MAST HA matrix or collagen powder + PRF	OLTs	48	24 months	Clinical improvement Regenerated tissue in various degree of remodeling, none had complete hyaline-like features at histology
PBSCs	Fu [55] 2014 Knee	Case report	Surgical delivery	PBSCs + autologous Periosteal flap + patellofemoral realignment	Knee chondral defects	1	7.5 years	Patient returned to competitive kickboxing Smooth surface 8 months after surgery Significant clinical and MRI improvements
	Turajane [53] T 2013 J Med Assoc Thai	Case series	IA injection	PBSCs + GFs addition/preservation + HA + microdrilling	Knee OA	5	6 months	Improvement in all clinical scores without adverse events
	Saw [54] 2013 Arthroscopy	RCT	IA injection	Subchondral drilling PBSCs + HA vs. HA 5 IA injections post-op 3 more IA injections after 6 months	Knee chondral defects	25 drilling + (PBSCs + HA) 25 drilling + HA	2 years	Comparable significant clinical improvement for both groups PBSCs + HA had both MRI and histology superior vs. control group
	Skowronski [56] 2012 Orthop Traumatol Rehabil	Case series	Surgical delivery	PBSCs covered by collagen membrane	Knee chondral defects	52	6 years	No adverse events Improvement in all clinical scores at 12 months. Poor outcomes in 2 patients at 12 months At 72 months minor deterioration in 2 more patients
	Saw [52] 2011 Arthroscopy	Case series	IA injection	Subchondral drilling + 5 weekly IA injections	Knee chondral defects	5	10–26 months	No adverse events hyaline cartilage regeneration at histology
BMC vs. PBSCs	Skowronski [77] 2013 Orthop Traumatol Rehabil	Comparative	Surgical delivery	PBSCs vs. BMC covered by collagen membrane	OLKs	21 BMC 25 PBSCs	5 years	Superior results in PBSCs group: good cartilaginous surface and integration. Slight clinical scores decrease in both groups at 60 months
SDSCs	Sekiya [76] 2015 Clin Orthop Relat Res	Case series	Surgical delivery	Cultured cells Scaffold free	Knee chondral defects	10	48 months	Significant clinical improvement Positive findings at MRI, and hyaline like in 3/4 at histology
ADSCs	Kim [71] 2015 AJSM	Comparative	Surgical delivery vs. IA injection	Subcutaneous fat SVF on FG scaffold vs. PRP-SVF injection	Isolated focal defects in knee OA	20 SVF-FG 20 SVF-PRP	28.6 months	Significant improvement in both groups. Better clinical results at final f-up and 2nd look appearance at 12 months for SVF-FG. No. of cells correlated with outcomes only for injective group
	Kim [72] 2015 Osteoarthritis Cartilage	Case series	Surgical delivery	Subcutaneous fat SVF + FG scaffold	Isolated focal defects in OA knee	20	27.9 months	Significant clinical and MRI scores improvement MRI correlates with clinical outcomes
	Michalek [67] 2015 Cell Transplant	Case series	IA injection	Subcutaneous fat SVF	OA (various joints)	1114	17.2 months	No adverse effects, safe, cost-effective Clinical improvement at 3–12 months. Follow-up at 12 months: 63 % patients had ≥75 % score improvement 91 % patients had ≥50 % score improvement Slower healing for obese and worse OA
	Koh [74] 2015 Arthroscopy	RCT	Surgical delivery	Subcutaneous fat MFX + FG + SVF vs. MFX	Knee chondral defects	40 MFX + SVF-FG 40 MFX	27.4 months	KOOS pain and symptoms better for SVF vs. control 2nd look: complete coverage 65 vs. 45 % SVF better MRI scores
	Kim [73] 2015 AJSM	Case series	Surgical delivery	Subcutaneous fat SVF + FG	Isolated Focal defects in OA knee	49	26.7 months	74.5 % good/excellent results Patient age >60 years or lesion size >6.0 cm^2^ are predictors of clinical failure
	Jo [59] 2014 Stem cell	Case series	IA injection	Cultured subcutaneous Phase I: low dose (1.0 × 10^7^) vs. mid-dose (5.0 × 10^7^) vs. high dose (1.0 × 10^8^) Phase II: 18 patients received only high dose	Knee OA	Phase I: 9 Phase II: 18	6 months	High-dose was more effective for knee function improvement MRI: decreased defect size and improved cartilage volume No adverse events related to cell dose
	Kim [69] 2014 AJSM	Comparative	IA injection	Subcutaneous fat SVF + marrow stimulation vs. marrow stimulation	OLTs	24 marrow stim + SVF 26 marrow stimulation	21.9 months	All clinical and MRI scores in SVF group improved significantly with respect to marrow stimulation alone SVF gave better outcomes for patients older than 46.1 years, lesion size >152.2 mm^2^, or in presence of subchondral cysts
	Kim [71] 2014 AJSM	Comparative	Surgical delivery	Subcutaneous fat SVF local adherent vs. SVF + FG	Isolated focal defects in OA knee	17 FG 37 scaffold-free	28.6 months	Both comparable clinical improvement 2nd look arthrosocopy at 12.3 months f-up: better ICRS scores for FG group
	Bui [62] 2014 Biomed Res Ther	Case series	IA injection	Subcutaneous fat SVF + PRP	Knee OA	21	8.5 months	Significant clinical scores improvement. No side effects. MRI: increased cartilage thickness
	Koh [70] 2014 AJSM	Case series	Surgical delivery	Subcutaneous fat SVF	Isolated focal defects in knee OA	35	26.5 months	Clinical improvement 76 % abnormal repair tissue at 2nd look arthroscopy (12.7 months f-up) Better outcomes if size <5.4 cm^2^ and/or BMI < 27.5
	Koh [74] 2014 Arthroscopy	RCT	IA injection	Subcutaneous fat HTO + PRP vs. HTO + PRP + SVF	Knee OA	23 HTO + PRP + SVF 21 HTO + PRP	24 months	SVF produced better improvement of KOOS pain and symptoms and VAS pain Fibrocartilage coverage SVF 50 vs. 10 % control
	Pak [61] 2013 BMC Musculoskelet Disord	Case series	IA injection	Subcutaneous fat SVF + PRP	OA (various joints)	91	26.7 months	SVF/PRP injections are safe Clinical improvement knee and hip
	Kim [68] 2013 AJSM	Comparative	IA injection	Subcutaneous fat SVF + PRP	Isolated defect in ankle OA	35 MFX 30 MFX + SVF	21.8 months	Clinical improvement both groups SVF group better results, especially applied to Tegner score Large lesion and/or subchondral cysts affected outcomes only for MFX alone
	Koh [65] 2013 KSSTA	Case series	IA injection	Subcutaneous fat SVF + PRP	Knee OA	30	24 months	Significant clinical improvement 14/16 (87.5 %) of 2nd look arthroscopy within 24 months improved or maintained cartilage status. Further clinical improvement 24 vs. 12 months
	Koh [64] 2013 Arthroscopy	Case series	IA injection after debridement	Fat pad SVF + PRP	Knee OA	18	24 months	function and pain improvement. Womac and MRI correlate with cell no. Better if OA ≤ 3
	Koh [63] 2012 Knee	Comparative	IA injection After debridement	Fat pad SVF + PRP	Knee OA	25 debridement + SVF-PRP 25 debridement	12 months min	Both improved scores. SVF performed better in <55 years and OA ≤ 3 (ICRS)
	Pak [60] 2011 J Med Case Rep	Case report	IA injection	Subcutaneous fat SVF + PRP + low dose dexamethasone	Knee OA	2	3 months	Clinical improvement Significant positive changes at MRI

### BMSCs

An increasing number of papers have been focused on this cell source in the past few years, both as BMSCs and BMC. Cultured BMSCs and BMC differ for composition, since adult bone marrow contains heterogeneous blood cells at various differentiation stages [13]. Thus, the harvest includes plasma, red blood cells, platelets, and nucleated cells, a small fraction of which contains adult MSCs that can be isolated through culture expansion [14]. However, even if not expanded, the heterogeneity of cell progenitor types in BMC might positively influence tissue regeneration [15]. Moreover, cell culture not only offers a higher number of cells but also presents high costs and some regulatory problems, since these products might be considered as pharmacological treatments by regulatory agencies. Thus, one-step techniques using BMC for the delivery of autologous cells in a single time are gaining increasing interest in the clinical setting. Besides these considerations, positive findings are leading the research towards the use of both cell-based strategies.

#### Cultured BMSCs: injective treatment

In 2008, Centeno and colleagues [16] first reported the promising clinical and MRI improvements at early follow-up after single intra-articular (i.a.) injection of autologous cultured BMSCs in a patient with knee degenerative cartilage disease, and similar findings at short term were later shown also by the groups of Davatchi [17], Emadedin [18], and Sol Rich [19]. Orozco et al. confirmed a rapid and progressive clinical improvement of knee OA in the first 12 months [20], which was maintained at 24-month follow-up, together with improved cartilage quality at MRI [21]. Finally, Davatchi et al. [22] updated their report, showing gradual mid-term deterioration of the outcomes in advanced OA.

Among comparative studies, Lee et al. [23] tested two administration strategies for focal knee cartilage defects and found no differences either by using BMSCs implantation under periosteum flap or microfractures (MFX) plus BMSCs i.a. injection, thus endorsing the less invasive approach.

Three randomized controlled trials (RCTs) have also been published. Wong et al. [24] treated knee unicompartmental OA with varus malalignment by combined high tibial osteotomy (HTO) and MFX. Patients randomly received post-operative i.a. injection of BMSCs-hyaluronic acid (HA) or HA alone as control. Both groups improved their scores, but BMSCs produced better clinical and MRI outcomes. Vangsness et al. [25] administered a single i.a. injection in patients after medial partial meniscectomy. Patients were randomized in two treatment groups (low- or high-dose allogeneic cultured BMSCs with HA) and a control group (HA-only). Both treatment groups showed improved clinical scores versus control, and MRI showed signs of meniscal volume increase at 24 months. Finally, Vega et al. [26] randomized two treatment groups for knee OA: a significantly greater improvement was shown after a single allogeneic BMSCs injection compared to control HA.

#### Cultured BMSCs: surgical delivery

Adachi et al. [27] observed cartilage and bone regeneration in a biopsy after cultured BMSCs implantation on hydroxyapatite-ceramic scaffold for osteochondral knee lesion (OLK). Haalem et al. [28] implanted BMSCs on a platelet fibrin glue (FG) scaffold, showing significant improvement and complete MRI filling of the cartilage defect. Kasemkijwattana et al. [29] seeded cells on a collagen scaffold with positive results in two traumatic knee lesions. Similarly, Kuroda et al. [30] had good results implanting BMSCs on collagen membrane with periosteum coverage in a judo-player knee, with hyaline-like tissue at a 12-month histology evaluation. Wakitani et al. used the same technique with positive findings also for patellofemoral lesions [31], stable at mid-term follow-up [32]. They also performed a comparative evaluation of this technique for focal defects in OA knees: two groups were treated with HTO, with or without BMSCs augmentation [33]. BMSCs-group showed better histology, but clinical scores comparable to the cell-free group. Nejadnik et al. compared BMSCs implantation with first-generation ACI in two groups of patients and observed comparable benefits [34].

Finally, Richter et al. [35] investigated the outcomes offered by BMSCs onto a collagen matrix for chondral ankle lesions, confirming no complications and a promising clinical improvement at 24 months of follow-up.

#### BMC: injective treatment

A single study by Varma et al. [36] reported promising results with BMC injection after arthroscopic debridement for knee OA, with increased benefits compared to debridement alone.

#### BMC: surgical delivery

The group of Giannini published several studies of scaffold-associated BMC implantation in knee and ankle joint defects. In their first study [37], they showed clinical and MRI improvements at 24 months after BMC implantation into collagen powder or HA matrix for osteochondral lesions of the talus (OLTs). Later [38], they reported a significant worsening between 24 and 48 months of follow-up, but the final result was still satisfactory compared to the basal level. Patients with longer symptoms before surgery had worse clinical outcomes. They also observed no degeneration progression at 24 months in five hemophilic ankle lesions [39], and similar results were confirmed in a larger group of patients treated for OLTs or ankle OA defects [40]. Also, this study showed a worsening trend after 24 months with a higher failure rate, which underlined the influence of OA degree and patient BMI. Moreover, a further study by Buda et al. [41] confirmed a similar trend of gradual worsening up to 72 months after scaffold-assisted BMC implantation.

Giannini et al. [42] also performed comparative evaluations: positive and similar clinical outcomes were found in three groups of patients treated with one-step BMC-HA matrix implantation versus open ACI or arthroscopic MACT for OLTs at 36 months of follow-up. These results were later confirmed at 48 months after collagen scaffold implantation, seeded either with BMC or cultured chondrocytes, with better tissue quality at MRI for the BMC group [43]. Moreover, a RCT by Cadossi et al. [44] highlighted that biophysical stimulation with pulsed electromagnetic fields (PEMFs) might improve the results at 12 months after collagen matrix-BMC implantation for OLTs.

Matrix-assisted BMC implantation was also investigated for the treatment of OLKs. The promising results using BMC on HA matrix were first reported by Buda et al. at short-term follow-up, with positive MRI and histology findings [45, 46], and then confirmed by Gobbi et al. [47], who observed superior outcomes using BMC instead of chondrocytes for the treatment of large patellofemoral defects. Similar results were obtained also by seeding BMC on collagen scaffolds: Gigante et al. [48] used BMC-enhanced AMIC technique with positive short-term clinical results, but limited tissue quality at histology [49], and Gobbi et al. [50] observed hyaline appearance and better short-term improvement in patients younger than 45 years and with single and smaller lesion. Finally, Skowronski et al. [51] documented stable mid-term outcomes after the treatment of large chondral lesions.

### PBSCs

The possibility of using autologous PBSCs obtained by culture expansion from a venous sample was first introduced by Saw et al. [52], who treated chondral knee lesions with subchondral drilling and five postoperative i.a. injections of PBSCs and HA, reporting no adverse reactions and positive histological findings. Turajane and colleagues [53] showed short-term clinical improvement using the same technique in early knee OA patients. Later, the group of Saw [54] also performed a RCT, documenting comparable clinical outcomes at 24 months, but better MRI and histological evaluations versus HA control.

With regard to surgical application, Fu et al. [55] reported optimal results at 7.5 years in a lateral trochlea lesion treated with patellar realignment plus periosteum-covered PBSCs implantation in a kick boxer, and Skowronski et al. [56] implanted PBSCs with a collagen membrane in a group of patients, reporting a stable improvement up to 72-month follow-up.

### ADSCs

ADSCs present a lower chondrogenic potential when compared with BMSCs [57]. Nonetheless, they can be obtained from liposuction, a simple and cheap procedure, and their clinical use is rapidly increasing, thanks to their easy availability and abundance [10]. Whereas the use of cultured cells has rarely been reported, the preferred technique involves cell harvest, collagenase digestion, and isolation of the stromal vascular fraction (SVF), a heterogeneous cell population that, among pre-adipocytes and immune cells, also includes ADSCs [58].

#### Injective treatment

Jo et al. [59] published the only available study on cultured ADSCs, applied at different doses: their preliminary clinical data showed no adverse events, and a clinical-MRI improvement at 6 months after injecting the highest dose.

Most of the literature focused instead on SVF. Regarding knee OA, Pak et al. [60] first obtained a promising clinical improvement 3 months after i.a. injection of subcutaneous SVF with HA, dexamethasone, and PRP in a patient. Later, they [61] confirmed safety and effectiveness of SVF injections in a larger cohort of patients treated into different joints. Bui et al. [62] also reported short-term clinical and MRI improvement after injection of SVF and PRP. However, the group of Koh was the main investigator of SVF use, starting from the infrapatellar fat pad source, in a case–control study [63]: all patients underwent debridement and the treatment group received an additional SVF-PRP injection. No major adverse events and a tendency for better outcomes were observed in the SVF group. The improvement was confirmed at 24 months in a further study [64]. The number of injected cells correlated with both clinical and MRI outcomes, while SVF had lower effects on the final stage OA. Later, the same group began to process subcutaneous fat with an analogous technique. They treated knee OA in elderly patients with arthroscopic lavage and SVF-PRP injection [65]: clinical improvement was obtained both at 12 and 24 months, and positive findings were reported at second look evaluation. Moreover, SVF injections significantly improved the benefits of high tibial osteotomy (HTO) for symptomatic varus knee, compared to control (HTO and PRP-only), both at clinical and second look evaluation [66].

Michalek et al. [67] administered single-dose SVF injections to the largest available group of patients, reporting no treatment-related adverse events and gradual clinical improvement between 3 and 12 months, with a slower recovery for obese and higher OA degrees.

Finally, the group of Koh also investigated SVF use in the ankle joint: Kim et al. injected SVF after marrow stimulation in two comparative studies, and observed higher clinical and MRI improvement both for ankle OA [68] or OLTs [69], compared to surgery alone. The benefit was greater for younger patients with smaller lesions, but the treatment was effective even in older patients.

#### Surgical delivery

Koh et al. [70] reported a significant clinical improvement 2 years after a scaffold-free SVF implantation for focal chondral lesions in OA knees, but abnormal repair tissue was observed in most cases at second look evaluation. In a subsequent study, the association with FG as scaffold significantly improved tissue quality, even though clinical results remained similar to SVF alone [71]. Later, they reported positive short-term results and correlation with MRI findings after SVF-FG implantation for OA [72]. Furthermore, a larger prospective study confirmed good/excellent results in 75 % patients at 24 months [73]. Interestingly, older age, higher BMI, and larger defect size were negative predictors in all these studies. SVF-FG augmentation also improved the outcome versus MF alone in an RCT, despite comparable histology findings [74].

Finally, a study on matched-paired groups found comparable clinical results but better ICRS macroscopic scores at 12 months for SVF surgical implantation versus injective delivery, whereas at the further follow-up, a significant clinical superiority was also obtained for surgical SVF delivery [75].

### SDSCs

SDSCs are a promising source of stem cells for cartilage tissue engineering, thanks to the greatest chondrogenic and lowest osteogenic potential among MSCs [57]. Sekiya et al. [76] reported promising results up to mid-term follow-up using SDMSCs scaffold-free implantation into single knee cartilage defects, with ¾ biopsies showing hyaline cartilage.

### Comparative studies

Skowronski et al. [77] performed the only clinical comparative study among stem cell types showing superior results with PBSCs rather than BMC under a collagen membrane for OLKs at 5-year follow-up.

## Discussion

This systematic research highlighted that the use of mesenchymal precursors as a biological approach to treat cartilage lesions and OA has widely increased (Fig. 2), as confirmed by the growing number of clinical trials published on this topic. In addition to an intensive preclinical research, the use of these procedures has recently broken down the barriers towards clinical application, with more than half of the available papers published in the last 3 years. Different sources have been investigated for clinical application, especially targeting knee or ankle cartilage disease. Among them, the most exploited cell types are those derived from bone marrow and adipose tissue. Cells have been used either after culture expansion or simply concentrated for one-step procedures: in particular, adipose cells have been applied mainly through cell concentration, and cells derived from bone marrow are currently applied both after expansion or concentration, while PBSCs and SDSCs can be only exploited through in vitro expansion due to their low number.

Regardless of cell source and manipulation, cells have being administered either surgically or through i.a. injection, to target focal lesions as well as degenerative joint disease.

Overall, despite the increasing literature on this topic, there is still limited evidence about the use of MSCs for the treatment of articular cartilage, in particular as far as high-level studies are concerned: in fact, most of the available papers are case series, while only few papers reported RCTs. Moreover, the few high level studies do not allow to clearly prove the effective potential of MSCs, due to the limited number of patients treated and to the presence of several confounding factors (PRP concomitant use, cell use in combination with scaffolds, etc.). To this regard, while several studies applied cells in association with PRP, with the rationale to provide both cells and growth factors at the same time, there is no evidence that adding platelet-derived growth factors provides any increased benefit with respect to cell administration alone, and specifically designed studies are needed in order to clarify the role of PRP with respect to MSCs and/or scaffolds in cartilage treatment. Furthermore, the tissue harvest procedure poses practical and ethical limitations which prevent from performing studies with a blinded design, therefore leaving an important bias related to the placebo effect, which is an important issue in this field of new fashionable regenerative treatments.

On the other hand, the available studies still allow to draw some indications on potential and limitations of MSCs clinical use for the treatment of cartilage lesions and OA.

First, the use of MSCs in the clinical setting can be considered safe, since no major adverse events related to the treatment nor to the cell harvest have been reported, at least from the available reports at short- to mid-term follow-up. Second, a clinical benefit of using MSCs therapies has been reported in most of the studies, regardless of cell source, indication, or administration method. This effectiveness has been reflected by clinical improvement but also positive MRI and macroscopic findings, whereas histologic features gave more controversial results among different studies. Third, different studies also gave a few indications regarding the patients who might benefit more from MSCs treatment: young age, lower BMI, smaller lesion size for focal lesions, and earlier stages of OA joints have been shown to correlate with better outcomes, even though the available data strength does not allow to define clear cutoff values.

The systematic analysis of the literature also allowed to underline other interesting findings that deserve to be discussed. Definite trends can be observed with regard to the delivery method: while different combinations of products and delivery methods have been investigated over the years, currently cultured cells are mostly being administered by i.a. injection, while one-step surgical implantation is preferred for cell concentrates. The different trends observed in this field are explained both by the controversial preclinical and clinical findings, which still leaves space for clinical investigations in opposite direction, but also by practical considerations, both in terms of economical, ethical, and regulatory limitations [6]. Many aspects are taken in consideration for the treatment choice, with physicians and researchers exploring different strategies, each one presenting potential advantages and possible drawbacks. To this regard, while culture expansion guarantees a selected MSC lineage to be delivered, but presenting high costs and some contamination risks related to cell manipulation, cell concentration offers a lower number of MSCs, in a heterogeneous cell population, and can be performed in one step, thus simplifying the procedure, reducing costs, and increasing patient compliance. To date, no clear evidence of superior outcome between the two cell manipulations is available, and also their most effective delivery method remains to be defined, with only a single retrospective study reporting better results for surgical delivery compared to i.a. SVF injection in a matched-paired analysis of two groups treated for single focal defects in knee OA [75]. Regarding surgical implantation, the use of solid scaffolds has been shown to be beneficial for SVF implantation [71], and it is the gold standard for the application of BMC [37–41, 43, 47, 48, 50, 51]. The good results obtained with scaffolds implanted with BMC have been compared with chondrocyte-based surgical techniques, showing similar outcomes, but with the advantage of the one-step approach [42, 43, 47].

Finally, regardless of cell source, manipulation and delivery method, the optimal cell dose is still under investigation. After a first preliminary study reported no complications related to high dose of cultured ADSCs [59], only a single clinical study specifically focused on this aspect, suggesting benefits and absence of side effects by using higher dose of BMSCs for the treatment of post-meniscectomized knees [25]. However, the lack of standardization and the heterogeneity of the studies reported in the current literature do not allow to extend these findings to the several proposed MSCs treatment strategies.

The clinical application of MSCs for the treatment of articular cartilage defects and OA shows promising results, but too many questions still remain open. Even though no complications have been reported, longer follow-ups on broader patient population are needed to confirm the safety of these procedures. Likewise, while promising results have been shown, the potential of these treatments should be confirmed by reliable clinical data through double-blind, controlled, prospective, and multicenter studies with longer follow-up. In addition, specific studies should be designed to identify the best cell sources, manipulation, and delivery techniques, as well as pathology and disease phase indications, with the aim of optimizing the outcome for a treatment focused on focal chondral defects or joint degeneration.

## Conclusions

This systematic review revealed a high interest of researchers in the clinical use of MSCs for cartilage and OA treatment, as testified by the increasing number of reports published over time. Whereas the lack of contraindication and generally promising clinical outcomes have been reported, the prevalence of low-quality studies, with many variables, shows several aspects that still need to be optimized, such as the best cell source and the most appropriate processing method, the most effective dose and delivery procedure. On the other hand, the first hints on the kind of patients who might benefit more from these procedures are being drawn. High-level studies with large number of patients and long-term follow-up are mandatory to evaluate the real potential of this biological approach for cartilage repair.

## Abbreviations

ADSCs: mesenchymal stem cells derived from adipose tissue
BMC: bone marrow concentrate
BMSCs: bone marrow expanded stem cells
FG: fibrin glue
HA: hyaluronic acid
HTO: high tibial osteotomy
i.a.: intra-articular
MAST: matrix-assisted stem cells transplantation
MFX: microfractures
MSCs: mesenchymal stem cells
OA: osteoarthritis
OLK: osteochondral knee lesion
OLTs: osteochondral lesions of the talus
PBSCs: stem cells derived from peripheral blood
PEMFs: pulsed electromagnetic fields
RCT: randomized controlled trial
SDSCs: mesenchymal stem cells derived from synovial tissue
SVF: stromal vascular fraction
